# Dynamic Interleukin-6 Level Changes as a Prognostic Indicator in Patients With COVID-19

**DOI:** 10.3389/fphar.2020.01093

**Published:** 2020-07-17

**Authors:** Zeming Liu, Jinpeng Li, Danyang Chen, Rongfen Gao, Wen Zeng, Sichao Chen, Yihui Huang, Jianglong Huang, Wei Long, Man Li, Liang Guo, Xinghuan Wang, Xiaohui Wu

**Affiliations:** ^1^ Department of Plastic Surgery, Zhongnan Hospital of Wuhan University, Wuhan, China; ^2^ Department of Thyroid and Breast Surgery, Zhongnan Hospital of Wuhan University, Wuhan, China; ^3^ Department of Rheumatology and Immunology, Tongji Hospital, Tongji Medical College, Huazhong University of Science and Technology, Wuhan, China; ^4^ Department of Ophthalmology, Zhongnan Hospital of Wuhan University, Wuhan, China; ^5^ Department of Urology, Zhongnan Hospital of Wuhan University, Wuhan, China; ^6^ Department of Neurosurgery, Zhongnan Hospital of Wuhan University, Wuhan, China

**Keywords:** COVID-19, interleukin-6, SARS-CoV-2, CT scores, infectious disease, lung injury

## Abstract

**Background:**

Interleukin-6 (IL-6), a proinflammatory cytokine, has been reported to be associated with disease severity and mortality in patients with coronavirus disease 2019 (COVID-19). Yet, dynamic changes in IL-6 levels and their prognostic value as an indicator of lung injury in COVID-19 patients have not been fully elucidated.

**Objective:**

To validate whether IL-6 levels are associated with disease severity and mortality and to investigate whether dynamic changes in IL-6 levels might be a predictive factor for lung injury in COVID-19 patients.

**Methods:**

This retrospective, single-center study included 728 adult COVID-19 patients and used data extracted from electronic medical records for analyses.

**Results:**

The mortality rate was higher in the elevated IL-6 group than in the normal IL-6 group (0.16 *vs* 5%). Cox proportional hazards and logistic regression analyses for survival (adjusted hazard ratio, 10.39; 95% confidence interval [CI], 1.09–99.23; *p* = 0.042) and disease severity (adjusted odds ratio, 3.56; 95% CI, 2.06–6.19; *p* < 0.001) revealed similar trends. Curve-fitting analyses indicated that patient computed tomography (CT) scores peaked on days 22 and 24. An initial decline in IL-6 levels on day 16 was followed by resurgence to a peak, nearly in tandem with the CT scores.

**Conclusion:**

Increased IL-6 level may be an independent risk factor for disease severity and in-hospital mortality and dynamic IL-6 changes may serve as a potential predictor for lung injury in Chinese COVID-19 patients. These findings may guide future treatment of COVID-19 patients.

## Introduction

The outbreak of coronavirus disease (COVID-19), first detected in Wuhan, China, in December 2019, has been categorized as a pandemic since March 2020. As COVID-19 continues to spread worldwide, 5,722,859 cases and 356,279 deaths have been reported as of May 28, 2020.

The Leishenshan (Thunder God Mountain) Hospital, the largest makeshift hospital in China, played an essential role in the battle against COVID-19 in Wuhan, China. The hospital had the capacity to accommodate 1,600 patients and was well equipped with intensive care units and operating rooms as well as clinical laboratories providing relatively standardized and systematic medical information on COVID-19 patients.

Interleukin 6 (IL-6) and other components of the inflammatory cascade contribute to host defense against infections. However, excessive synthesis of IL-6 can lead to a severe acute systemic inflammatory response known as a “cytokine storm,” which confers increased risks of vascular hyperpermeability, multiorgan failure, and eventually death (Meduri et al., 1995). This pathophysiological process, which is supported by many studies, suggests that higher IL-6 levels may be an important predictor of COVID-19 severity (Cai et al., 2020; Chen et al., 2020). However, the role of IL-6 as an independent predictor of severity and mortality in hospitalized COVID-19 patients has not been validated.

Acute respiratory distress syndrome (ARDS), one of the major factors contributing to mortality in COVID-19 patients, is similar to severe community-acquired pneumonia caused by other viruses or bacteria (Ranieri et al., 2012; Jose and Manuel, 2020). The pathophysiological process of ARDS involves a cytokine storm caused by the overproduction of early response proinflammatory cytokines. Theoretically, monitoring the IL-6 level is crucial to understanding the pathophysiological process of ARDS; however, whether IL-6 levels can predict the severity of pulmonary injury has not been fully investigated in patients with COVID-19.

Thus, in this study, we aimed to determine whether IL-6 levels are associated with disease severity and mortality in COVID-19 patients and investigate whether dynamic changes in IL-6 levels are predictive of pulmonary lesions caused by ARDS in hospitalized COVID-19 patients.

## Methods

### Patient Selection

This retrospective study included 1,880 confirmed COVID-19 patients who were admitted to the Leishenshan Hospital in Wuhan, China, between February 9 and April 9, 2020. Exclusion criteria were patients: younger than 18, missing data on IL-6 levels, acute myocardial infarction during hospitalization, acute pancreatitis, or transferred to another hospital. The dataset included in the final analysis consisted of 728 cases (**Figure S1**).

### Data Collection and Primary Outcomes

Detailed patient information, including demographic characteristics, history of comorbidities, symptoms, laboratory findings, computed tomography (CT) findings, length of hospital stay, oxygen support, illness severity on admission, and treatment, was collected from the electronic patient records stored in the hospital information management system and entered into a customized Excel form (Microsoft, Redmond, WA, USA).

The primary outcomes were patient survival and COVID-19 severity during hospitalization. Based on the 7^th^ Interim Guidance for the Diagnosis and Treatment of COVID-19 published by the National Health Commission of China, each patient was classified as mild, common, severe, or critical.

### Evaluation of CT Images

As chest CT has a high sensitivity for the diagnosis of COVID-19, studies have advocated for the use of CT images to evaluate the severity of acute respiratory syndrome (Wong et al., 2004; Ai et al., 2020; Lyu et al., 2020). Furthermore, radiographic scores have been shown to play an important role in the diagnosis of severe acute respiratory syndrome (SARS) between November 2002 and August 2003 (Wong et al., 2004). In our study, chest CT images were independently inspected by two radiologists who were blinded to the patients’ clinical data. An semi-quantitative scoring system, based on previous studies and the characteristics of COVID-19, was adopted for the assessment of pulmonary inflammation. Score 1 was generated based on CT imaging features such as ground-glass opacities (GGOs), reticulate or cord-like changes, consolidation, and pleural effusion. Each imaging feature received 1 point. While Score 1 comprised the sum of the points, Score 2 was generated based on the area of lung lobe involvement and graded from 0 to 4 as follows: no involvement, 0; <25% involvement, 1; 26–50% involvement, 2; 51–75% involvement, 3; and 76–100% involvement, 4. The overall score was the sum of Scores 1 and 2.

### Statistical Analyses

Patients were assigned to either the elevated or normal IL-6 group based on an IL-6 cutoff value of 7 pg/ml (obtained from medical records). Continuous variables are presented as medians and interquartile ranges (IQRs), whereas categorical variables are expressed as the number of patients (-percentages).

The Kruskal–Wallis or Mann–Whitney *U* test was used for intergroup comparisons between the elevated IL-6 and normal IL-6 groups with regard to the means of continuous variables. For categorical variables, intergroup comparisons were performed using the chi-squared test or Fisher’s exact test.

Cox proportional hazards regression models were used to determine the effect of IL-6 levels on overall survival. We used a multivariate Cox proportional hazards regression model adjusted for variables such as age, history of cardiovascular disease, lymphocyte count, D-dimer level, and lactate dehydrogenase level. Survival analysis was conducted using Kaplan–Meier survival curves and the log-rank test or Mantel–Haenszel test. Moreover, the cumulative hazard function for COVID-19 progression in both groups was analyzed. The relationship between IL-6 levels and CT scores of lung injury was assessed using a curve-fitting analysis. Receiver-operating characteristic (ROC) curve analysis was used to evaluate the predictive ability for death and disease severity.

All statistical analyses were performed using R version 3.6.1 (https://www.r-project.org/, R Core Team, Vienna, Austria) and EmpowerStats version 2.0- (http://www.empowerstats.com/cn/, X&Y Solutions, Inc., Boston, MA). A two-sided *p*-value <0.05 was considered statistically significant.

### Ethics Approval and Informed Consent

The study protocol was approved by the Research Ethics Committee of the Zhongnan Hospital of Wuhan University (approval no.: 2020074). The Research Ethics Committee waived the need for informed consent from the patients due to the urgent need for research insights in the context of this rapidly evolving infectious disease. All procedures undertaken in this study were in accordance with the ethical standards of the institutional and national research committee as well as the 1964 Declaration of Helsinki and its later amendments and other comparable ethical standards.

## Results

### Baseline Characteristics of the Study Participants

The baseline characteristics of 728 patients are listed in **Table 1**. The median ages in the normal and elevated IL-6 groups were 57 and 68 years, respectively. A total of seven (0.96%) patients died. In the normal IL-6 group, the majority of patients (54.4%) were female. In contrast, there was a higher proportion (54.2%) of male patients in the elevated IL-6 group. Cardiovascular diseases were more prevalent in the elevated IL-6 group (44.17%) than in the normal IL-6 group (20.23%); similarly, neurological diseases were also more common in the elevated IL-6 than in the normal IL-6 group (11.76 *vs.* 4.93%). As shown in **Table 2**, the levels of routine blood test parameters, including leukocyte count, neutrophil count, were significantly higher in the elevated IL-6 group than in the normal IL-6 group. Moreover, the lymphocyte, erythrocyte, and platelet counts and hemoglobin concentration were reduced in the elevated IL-6 group. With regard to liver function parameters, the direct bilirubin level was higher and accompanied by notable reductions in albumin and total protein levels in the elevated IL-6 group. An evaluation of renal function showed significantly elevated serum creatinine, urea, and cystatin C levels. Lactate dehydrogenase levels were increased in the elevated IL-6 group. Significant abnormalities in blood coagulation parameters, such as prolonged prothrombin time and activated partial thromboplastin time, higher International Normalized Ratio, and increased D-dimer levels, were noted in the elevated IL-6 group. Moreover, the proportion (84.62%) of patients with positivity for immunoglobulin G (IgG) was lower in the elevated IL-6 group than in the normal IL-6 group (94.03%). The clinical treatment and outcome analyses revealed that more patients in the elevated IL-6 group were likely to require antibiotics, antiviral drugs, anticoagulants, corticosteroids, and vitamin C therapy. Furthermore, in the normal IL-6 group, there was a higher use of antimalarial drugs (83.93%) and traditional Chinese medicine (88.82%). Patients in the elevated IL-6 group were more likely to be admitted to the intensive care unit during hospitalization (20.00%) than patients in the normal IL-6 group (0.82%) (**Table 3**).

**Table 1:** Demographic characteristics and symptoms of 728 patients with COVID-19.

Covariates	Levels	Normal IL-6(n = 608), n (%)	Elevated IL-6(n = 120), n (%)	All patients(n = 728), n (%)	P value
age, median (IQR)		57.00 (48.00–66.00)	68 (59–75)	58 (49–68)	<0.001
gender					
	female	331 (54.4%)	55 (45.8%)	386 (53.02)	<0.001
	male	277 (45.6%)	65 (54.2%)	342 (46.98)	
					
Any comorbidity		171 (60.21%)	76 (74.51%)	247 (63.99%)	0.010
	Cardiovascular diseases	123 (20.23%)	53 (44.17%)	176 (24.18%)	<0.001
	Respiratory system diseases	25 (9.12%)	4 (5.26%)	29 (8.3%)	0.28
	Endocrine diseases	47 (16.55%)	16 (15.69%)	63 (16.3%)	0.84
	Malignancy	19 (6.69%)	10 (9.80%)	29 (7.5%)	0.306
	Digest system diseases	12 (4.23%)	9 (8.82%)	21 (5.4%)	0.079
	Neurological diseases	14 (4.93%)	12 (11.76%)	26 (6.7%)	0.018
Initial symptoms					
	Fever or fatigue	213 (80.08%)	75 (76.53%)	288 (79.12%)	0.461
	Respiratory symptoms	217 (81.58%)	82 (83.67%)	299 (82.14%)	0.644
	Digestive symptoms	29 (10.90%)	11 (11.22%)	40 (10.99%)	0.931
	Neurological symptoms	7 (2.63%)	4 (4.08%)	11 (3.02%)	0.473
	Other	9 (3.38%)	2 (2.04%)	11 (3.02%)	0.507
Death		1 (0.16%)	6 (5%)	7 (0.96%)	<0.001

**Table 2:** Serum biochemical parameters and blood coagulation test results of 728 patients with COVID-19.

Covariate		Normal IL-6 (n = 608)	Elevated IL-6 (n = 120)	All patients (n = 728)	P value	Reference range
		Median (IQR)/n (%)	Median (IQR)/n (%)	Median (IQR)/n (%)		
**Liver function parameters**						
Alanine aminotransferase, U/L		26.00 (16.00–42.00)	23.00 (13.00–38.50)	25.00 (16.00–42.00)	0.177	9–50
	9–50	471 (77.72%)	89 (74.79%)	560 (77.24%)	0.11	
	<9	24 (3.96%)	10 (8.40%)	34 (4.69%)		
	>50	111 (18.32%)	20 (16.81%)	131 (18.07%)		
Aspartate aminotransferase, U/L		21.00 (16.00–28.00)	22.00 (16.00–34.00)	21.00 (16.00–29.00)	0.062	15–40
	15–40	455 (75.08%)	71 (59.66%)	526 (72.55%)	<0.001	
	<15	97 (16.01%)	22 (18.49%)	119 (16.41%)		
	>40	54 (8.91%)	26 (21.85%)	80 (11.03%)		
Total bilirubin, μmol/L		9.10 (7.20–11.97)	9.10 (7.20–11.97)	9.20 (7.10–12.20)	0.16	5.0–21.0
	5–21	557 (91.91%)	100 (84.03%)	657 (90.62%)	<0.001	
	<5	34 (5.61%)	7 (5.88%)	41 (5.66%)		
	>21	15 (2.48%)	12 (10.08%)	27 (3.72%)		
Direct bilirubin, μmol/L		3.05 (2.40–4.00)	4.00 (2.80–5.95)	3.10 (2.40–4.30)	<0.001	0–7
	0–7	584 (96.37%)	97 (81.51%)	681 (93.93%)	<0.001	
	>7	22 (3.63%)	22 (18.49%)	44 (6.07%)		
Indirect bilirubin, μmol/L		5.70 (4.40–8.00)	5.05 (4.03–7.07)	5.60 (4.30–7.80)	0.049	1.5–18
	1.5–18	504 (98.63%)	99 (97.06%)	603 (98.37%)	0.231	
	<1.5	2 (0.39%)	0 (0.00%)	2 (0.33%)		
	>1.5	5 (0.98%)	3 (2.94%)	8 (1.31%)		
Total protein, g/L		66.50 (62.80–69.90)	62.70 (58.45–66.45)	65.90 (61.90–69.70)	<0.001	65–85
	65–85	375 (61.88%)	40 (33.61%)	415 (57.24%)	<0.001	
	<65	231 (38.12%)	79 (66.39%)	310 (42.76%)		
Albumin, g/L		38.0 (35.7–40.5)	34.2 (31.4–37.5)	37.70 (35.00–40.20)	<0.001	
	40–55	184 (30.36%)	9 (7.56%)	193 (26.62%)	<0.001	
	<40	422 (69.64%)	110 (92.44%)	532 (73.38%)		
						
**Renal function parameters**						
urea nitrogen, mmol/L		4.70 (3.80–5.70)	5.20 (4.00–6.95)	4.80 (3.80–5.80)	<0.001	2.7–7.6
	2.7–7.6	554 (91.57%)	85 (71.43%)	639 (88.26%)	<0.001	
	≤2.7	27 (4.46%)	10 (8.40%)	37 (5.11%)		
	>7.6	24 (3.97%)	24 (20.17%)	48 (6.63%)		
Creatinine, μmol/L		63.3 (53.7–74.7)	67.1 (54.2–84.6)	63.65 (53.77–75.60)	<0.001	64.0–104.0
	64–104	271 (44.79%)	46 (38.66%)	317 (43.78%)	<0.001	
	<64	314 (51.90%)	53 (44.54%)	367 (50.69%)		
	>104	20 (3.31%)	20 (16.81%)	40 (5.52%)		
Uric Acid,μmol/L		303.00 (245.00–367.00)	269.00 (199.00–357.50)	299.50 (237.75–366.00)	0.084	208–428
	208–428	506 (83.64%)	85 (71.43%)	591 (81.63%)	<0.001	
	<208	26 (4.30%)	20 (16.81%)	46 (6.35%)		
	>428	73 (12.07%)	14 (11.76%)	87 (12.02%)		
Cystatin C,mg/L		0.91 (0.80–1.01)	1.07 (0.92–1.36)	0.92 (0.82–1.06)	<0.001	0–1.2
	≤1.2	559 (92.40%)	79 (66.39%)	638 (88.12%)	<0.001	
	>1.2	46 (7.60%)	40 (33.61%)	86 (11.88%)		
**Cardiac enzyme parameters**						
Lactate dehydrogenase, U/L		184.00 (161.00–213.00)	225.15 (184.00–284.75)	189.00 (163.00–222.00)	<0.001	125–343
	125–343	546 (94.30%)	97 (85.09%)	643 (92.78%)	<0.001	
	<125	17 (2.94%)	1 (0.88%)	18 (2.60%)		
	>343	16 (2.76%)	16 (14.04%)	32 (4.62%)		
creatine kinase, ng/mL		53.0 (37.0–77.0)	44.4 (26.2–61.0)	51.00 (35.00–76.00)	<0.001	male ≤171 female ≤145
	Normal	551 (95.16%)	106 (92.98%)	657 (94.81%)	0.337	
	male >171, female >145	28 (4.84%)	8 (7.02%)	36 (5.19%)		
**Coagulation function parameters**						
Prothrombin time, s		11.3 (10.9–11.6)	11.8 (11.3–12.5)	11.35 (10.90–11.72)	<0.001	9.4–12.5
	9.4–12.5	546 (95.62%)	90 (76.92%)	636 (92.44%)	<0.001	
	<9.4	0	0	0		
	>12.5	25 (4.38%)	27 (23.08%)	52 (7.56%)		
Activated partial thromboplastin time, s		27.0 (24.3–30.3)	29.2 (26.8–33.0)	27.50 (24.70–30.50)	<0.001	25.1–36.5
	25.1–36.5	360 (63.05%)	86 (73.50%)	446 (64.83%)	<0.001	
	<25.1	188 (32.92%)	15 (12.82%)	203 (29.51%)		
	>36.5	23 (4.03%)	16 (13.68%)	39 (5.67%)		
Thrombin time, s		17.6 (17.0–18.4)	17.0 (16.1–17.9)	17.50 (16.90–18.33)	<0.001	10.3–16.6
	10.3–16.6	81 (14.19%)	42 (35.90%)	123 (17.88%)	<0.001	
	>16.6	490 (85.81%)	75 (64.10%)	565 (82.12%)		
International Normalized Ratio		0.97 (0.93–1.00)	1.02 (0.97–1.08)	0.97 (0.93–1.01)	<0.001	0.85–1.15
	0.85–1.15	566 (99.12%)	109 (93.16%)	675 (98.11%)	<0.001	
	>1.15	5 (0.88%)	8 (6.84%)	13 (1.89%)		
		2.90 (2.47–3.66)	3.73 (3.00–4.63)	3.00 (2.51–3.82)	<0.001	2.38–4.98
	2.38–4.98	381 (66.73%)	55 (47.01%)	436 (63.37%)	<0.001	
	<2.38	111 (19.44%)	13 (11.11%)	124 (18.02%)		
	>4.98	79 (13.84%)	49 (41.88%)	128 (18.60%)		
D-dimer, g/L		0.37 (0.21–0.82)	1.03 (0.56–2.61)	0.44 (0.23–1.04)	<0.001	0–0.50
	0-0.5	348 (60.95%)	28 (23.93%)	376 (54.65%)	<0.001	
	>0.5	223 (39.05%)	89 (76.07%)	312 (45.35%)		
						
**Routine blood tests**						
Leucocyte count, × 10⁹/L	WBC	5.66 (4.65–6.71)	6.46 (4.84–7.80)	5.7 (4.7–6.9)	<0.001	3.5–9.5
	3.5–9.5	550 (90.61%)	92 (76.67%)	642 (88.31%)	<0.001	
	<3.5	40 (6.59%)	10 (8.33%)	50 (6.88%)		
	>9.5	17 (2.80%)	18 (15.00%)	35 (4.81%)		
Neutrophil count, × 10⁹/L		3.18 (2.48–4.05)	4.23 (3.14–5.86)	3.29 (0.06–33.01)	<0.001	1.8–6.3
	1.8–6.3	533 (87.81%)	87 (72.50%)	620 (85.28%)	<0.001	
	<1.8	48 (7.91%)	7 (5.83%)	55 (7.57%)		
	>6.3	26 (4.28%)	26 (21.67%)	52 (7.15%)		
Lymphocyte count, × 10⁹/L		1.64 (1.33–1.98)	1.14 (0.81–1.52)	1.58 (0.17–3.56)	<0.001	1.1–3.2
	1.1–3.2	375 (61.78%)	44 (36.67%)	419 (57.63%)	<0.001	
	<1.1	39 (6.43%)	25 (20.83%)	64 (8.80%)		
	>3.2	193 (31.80%)	51 (42.50%)	244 (33.56%)		
Erythrocyte count, × 10^12^/L		4.13 (3.82–4.46)	3.66 (3.18–4.19)	4.08 (1.78–6.19)	<0.001	4.3–5.8
	4.3–5.8	207 (34.10%)	21 (17.50%)	228(31.36%)	<0.001	
	<4.3	397 (65.40%)	96 (80.00%)	493 (67.81%)		
	>5.8	3 (0.49%)	3 (2.50%)	6 (0.83%)		
Monocyte count, × 10⁹/L		0.50 (0.40–0.62)	0.56 (0.41–0.73)	0.50 (0.03–2.20)	0.032	0.1–0.6
	0.1–0.6	115 (18.95%)	34 (28.33%)	149 (20.50%)	0.005	
	<0.1	0 (0.00%)	1 (0.83%)	1 (0.14%)		
	>0.6	492 (81.05%)	85 (70.83%)	577 (79.37%)		
Hemoglobin, g/L		126.00 (116.00–137.00)	111.50 (98.75–122.25)	124.00 (50.00–178.00)	<0.001	130.0–175.0
	130.0–175.0	231 (38.06%)	21 (17.50%)	252 (34.66%)	<0.001	
	<130.0	376 (61.94%)	97 (80.83%)	473 (65.06%)		
	>175.0	0 (0.00%)	2 (1.67%)	2 (0.28%)		
Platelet count, × 10⁹/L		231.00 (192.00–277.00)	205.00 (151.00–270.75)	229.00 (187.00–277.00)	0.002	125.0–350.0
	125.0–350.0	541 (89.13%)	90 (75.00%)	631 (86.80%)	<0.001	
	<125.0	18 (2.97%)	16 (13.33%)	36 (4.95%)		
	>350.0	48 (7.91%)	14 (11.67%)	60 (8.25%)		
**Other serum tests**						
Procalcitonin, ng/ml		0.04 (0.02–0.05)	0.07 (0.05–0.13)	0.04 (0.03–0.06)	<0.001	<0.05
	<0.05	401 (71.4%)	27 (23.7%)	428 (63.31%)	<0.001	
	>=0.05	161 (28.6%)	87 (76.3%)	248 (36.69%)		
SARS-CoV-2 IgM					0.905	
	No	190 (65.97%)	45 (65.22%)	235 (65.83%)		
	Yes	98 (34.03%)	24 (34.78%)	122 (34.17%)		
SARS-CoV-2 IgG						
	No	16 (5.97%)	10 (15.38%)	26 (7.81%)	0.011	
	Yes	252 (94.03%)	55 (84.62%)	307 (92.19%)		

**Table 3:** Clinical treatments and outcomes of 728 patients with COVID-19.

Covariates	Levels	Normal IL-6 (n = 608) n (%)	Elevated IL-6 (n = 120) n (%)	All patients (n = 728)	P value
				n (%)	
Drugs					
	Antibiotic	149 (24.51%)	66 (55.00%)	215 (29.53%)	<0.001
	Antiviral drugs	271 (44.57%)	75 (62.50%)	346 (47.53%)	<0.001
	Antimalarial drugs	47 (83.93%)	5 (16.67%)	52 (60.47%)	<0.001
	Anticoagulants	30 (4.93%)	46 (38.33%)	76 (10.44%)	<0.001
	Corticosteroid	27 (4.44%)	23 (19.17%)	50 (6.87%)	<0.001
	Vitamin C	112 (18.42%)	36 (30.00%)	148 (20.33%)	0.004
	Traditional Chinese medicine	540 (88.82%)	98 (81.67%)	638 (87.64%)	0.03
Oxygen support					
	Low-flow nasal cannula	76 (85.39%)	21 (60.00%)	97 (78.23%)	0.002
	Non-invasive ventilation or high-flow nasal cannula	13 (14.61%)	10 (28.57%)	23 (18.55%)	<0.001
	Invasive mechanical ventilation	0 (0.00%)	3 (8.57%)	3 (2.42%)	<0.001
	ECMO	0 (0.00%)	1 (2.86%)	1 (0.81%)	<0.001
CT scores	1–4	27 (51.92%)	20 (40.00%)	47 (46.08%)	0.227
	5–7	25 (48.08%)	30 (60.00%)	55 (53.92%)	
Disease progression					
	Stableness/Hospitalization	3 (0.50%)	3 (2.73%)	6 (0.84%)	<0.001
	Improvement/Recover	601 (99.34%)	101 (91.82%)	702 (98.18%)	
	Death	1 (0.17%)	6 (5.45%)	7 (0.98%)	
Length of hospital stay, d, median (IQR)		20.0 (14.0–25.0)	20.0 (15.0–28.0)	20.00 (14.00–26.00)	0.243
ICU care		5 (0.82%)	24 (20.00%)	29 (93.55%)	0.007
Severity on admission					
	Mild	117 (19.24%)	20 (16.67%)	137 (18.82%)	<0.001
	General	365 (60.03%)	44 (36.67%)	409 (56.18%)	
	Severe	122 (20.07%)	43 (35.83%)	165 (22.66%)	
	Critical	4 (0.66%)	13 (10.83%)	17 (2.34%)	
Severity at worst					
	Mild	0	0	0	<0.001
	General	325 (53.5%)	18 (15.0%)	343 (47.18%)	
	Severe	273 (45.0%)	76 (63.3%)	349 (48.01%)	
	Critical	9 (1.5%)	26 (21.7%)	35 (4.81%)	

#### Univariate and Multivariate Analyses in Patients With Elevated IL-6 Levels

As shown in **Table 4**, we conducted a univariate analysis to identify whether an elevated IL-6 level was significantly associated with mortality (hazard ratio [HR], 30.54; 95% confidence interval [CI], 3.68–253.74; *p* < 0.002). In the next-step multivariate Cox regression analysis, IL-6 was identified as a significant indicator of mortality (HR, 10.39; 95% CI, 1.09–99.23; *p* = 0.042). Furthermore, the ordinal logistic regression model indicated that IL-6 was associated with disease severity in both the univariate (OR, 9.33; 95% CI, 5.72–15.22; *p* < 0.001) and multivariate (OR, 3.56; 95% CI, 2.06–6.19; *p* < 0.001) analyses (**Table 5**).

**Table 4:** The risk of elevated IL-6 for mortality of COVID-19.

Group		Cox Regression Analysis			
		HR	95% CI		P value
Univariate Analysis	IL-6 ≤7 pg/ml	ref			
	IL-6 >7 pg/ml	30.54	3.68	253.74	0.002
Multivariate Analysis*	IL-6 ≤7 pg/ml	ref			
	IL-6 >7 pg/ml	10.39	1.09	99.23	0.042

*Adjusted for age, history of cardiovascular disease, lymphocyte count, D-dimer, and lactate dehydrogenase.

**Table 5:** The risk of elevated IL-6 for disease severity of COVID-19.

Group		Logistics Regression Analysis			
		OR	95% CI		P value
Univariate Analysis	IL-6 ≤7 pg/ml	ref			
	IL-6 >7 pg/ml	9.33	5.72	15.22	<0.001
Multivariate Analysis*	IL-6 ≤7 pg/ml	ref			
	IL-6 >7 pg/ml	3.56	2.06	6.19	<0.001

*Adjusted for age, history of cardiovascular disease, lymphocyte count, D-dimer, and lactate dehydrogenase.

#### Mortality and Illness Severity in Patients With Elevated IL-6 Levels

**Figure 1:**
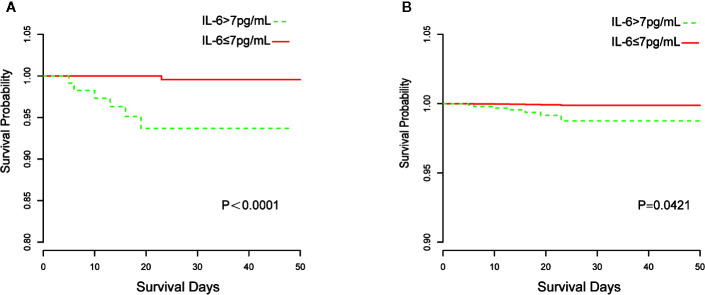
Kaplan-Meier curves of IL-6 group stratification for survival. **(A)** Without adjustment; **(B)** Adjusted for age, the history of cardiovascular disease, lymphocyte count, D-dimer, and lactate dehydrogenase.

**Figure 2:**
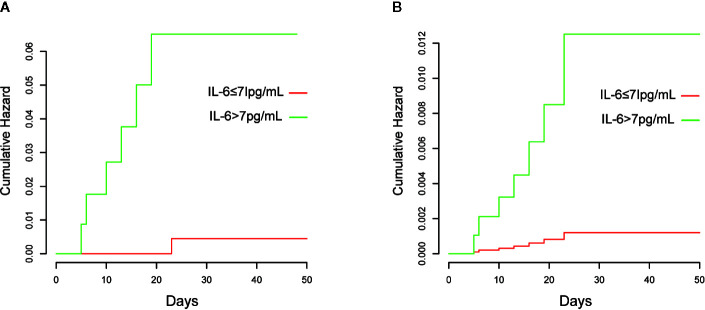
Cumulative hazards of death are presented. **(A)** Without adjustment; **(B)** Adjusted for age, history of cardiovascular disease, lymphocyte count, D-dimer, and lactate dehydrogenase.

**Figure 3:**
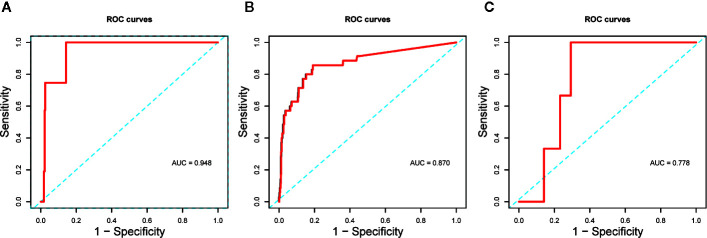
**(A)** ROC curve assessed the predictive capability of elevated IL-6 for death; **(B)** ROC curve assessed the predictive capability of elevated IL-6 for disease severity; **(C)** ROC curve assessed the predictive capability of elevated IL-6 for CT scores.

The Kaplan–Meier curves and cumulative hazard function showed that patients with normal IL-6 levels had better survival (*p* < 0.05, **Figures 1** and **2**). Further, the ROC analysis showed that the elevated IL-6 group had the largest areas under the ROC curve (AUCs) of 0.870 and 0.778 for disease severity and CT scores, respectively (**Figure 3**).

#### Curve Fitting and Trend Analyses for IL-6 Levels in COVID-19 Patients

**Figure 4:**
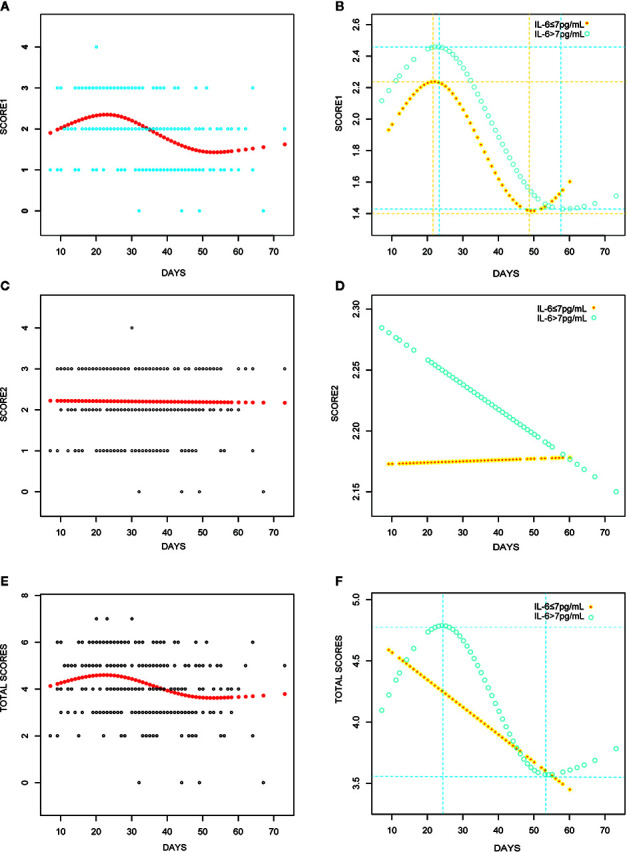
Curve fitting analyses with CT scores for all COVID-19 patients **(A, C, E)**; IL-6 elevated group (IL>7 pg/ml) *versus* IL-6 elevated group (IL≤ 7 pg/ml) **(B, D, F)**.

The peak value of Score 1 for all patients was 2.35 on day 23 (**Figure 4A**); however, while the peak value for the elevated IL-6 group was 2.46 on day 23, the peak value in the normal IL-6 group was 2.24 on day 22 (**Figure 4B**). Nonetheless, the peak value of Score 1 was attained within the same period in both groups. The Score 2-fitted curve for all patients presented a linear line that indicated a decline over time (**Figure 4C**). In the normal IL-6 group, the fitted curve presented a more uniform declining linear line than that in the elevated IL-6 group (**Figure 4D**). For the overall score, all patients showed peak values on day 22 (4.60; **Figure 4E**). Patients in the elevated IL-6 group showed the peak score on day 24 (4.79; **Figure 4E**), whereas a fitted curve that presented a flattened, declining, linear line was seen for patients in the normal IL-6 group.

**Figure 5:**
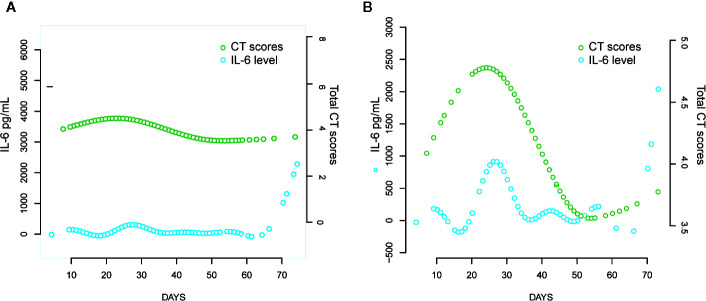
Comparison of CT scores trend and IL-6 levels over time with curve fitting analyses. **(A)** All the COVID-19 patients; **(B)** Patients with elevated IL-6.

The curve-fitting analysis was used to evaluate the changes in IL-6 levels: there was a slight decrease on day 16 and an increase to the peak level on day 26 (**Figure 5A**). A similar trend and an obvious dynamic change in IL-6 levels were observed in the elevated IL-6 subgroup (**Figure 5B**).

## Discussion

Using data from a unique makeshift hospital for COVID-19 patients, this results from this retrospective study showed that higher serum levels of IL-6 was an independent and reliable risk factor for COVID-19 patients and led to higher disease severity and mortality. Moreover, the trend fluctuation occurred earlier than the negative changes in lung CT scores, which indicated a potential predictive ability for lung injury. These findings provide clinical evidence in support of recently published guidance statements by Aziz et al. (2020). Nevertheless, due to the programmatic, retrospective nature of this research, the inferences derived from the data require further validation from cohort studies and randomized controlled trials (RCTs).

IL-6, a chemokine secreted by T-cells and macrophages to stimulate the immune response, is an important biomarker of inflammation, and elevated IL-6 levels have been demonstrated in inflammatory states induced by several pathological conditions (Cai et al., 2020). In COVID-19 patients, overproduction of cytokines, such as IL-6, might activate coagulation pathways, with a resultant disruption of procoagulant–anticoagulant homeostasis, induction of disseminated intravascular coagulation, and multiorgan dysfunction or failure (Jose and Manuel, 2020). In this study, we found that COVID-19 patients with elevated IL-6 levels had a higher likelihood of experiencing renal, hepatic, and lung injuries (**Table 2**).

Previous clinical studies have shown that older age, male sex, cardiovascular diseases, lower lymphocyte counts, higher D-dimer levels, and increased lactate dehydrogenase levels are significant risk factors for illness severity or mortality in COVID-19 patients (Li et al., 2020; Wang et al., 2020). In this study, there were a larger proportion of patients with these in the elevated IL-6 group. Thus, patients with high IL-6 levels may have a worse prognosis. Research has demonstrated that a higher IL-6 level on admission can predict greater odds of complications and illness severity in COVID-19 patients (Liu et al., 2020); however, mortality was not evaluated. Although a few studies have indicated that increases in IL-6 levels are potentially associated with an increased likelihood of mortality, they lacked statistical power as residual confounders were not considered or only indirectly validated through lower mortality after using an interleukin-6 receptor antagonist for blockading cytokine release in severe COVID-19 patients (Alattar et al., 2020; Aziz et al., 2020; Zhang et al., 2020). A multivariate Cox regression model was developed in our study to adjust for confounders including older age, history of cardiovascular disease, lower lymphocyte count, higher D-dimer levels, and increased lactate dehydrogenase levels. These risk factors of mortality have been validated in previous research (Ji et al., 2020; Li et al., 2020; Zhou et al., 2020). Additionally, in our study, Kaplan–Meier and cumulative hazard analyses were conducted to verify the strong associations of IL-6 levels with disease severity and mortality. Thus, all analyses conducted in our study validated that elevated IL-6 levels are an independent risk factor for the severity and mortality of COVID-19.

Furthermore, we evaluated the potential predictive function of IL-6. COVID-19 is caused by severe acute respiratory syndrome coronavirus 2 (SARS-CoV-2) (Huang et al., 2020), which binds to the alveolar epithelial cells to subsequently activate innate and adaptive immune responses and induces the release of a large number of cytokines, including IL-6. Moreover, considering the inherent role of these proinflammatory factors, vascular permeability increases, followed by the influx of large amounts of fluid and a large number of blood cells into the alveoli, resulting in dyspnea or ARDS (Knudsen and Ochs, 2018; Leiva-Juarez et al., 2018). The flux in IL-6 levels is associated with the pathological processes that mediate lung injuries, which was demonstrated by the similarity in peak timings of IL-6 levels and CT scores (**Figure 4**) in this study. Interestingly, IL-6 levels showed a slight decrease prior to the increase in CT scores in the fitting curve. This may be a result of SARS-CoV-2 eliciting attenuated innate immunity, leading to delayed proinflammatory cytokine induction, similar to the reaction observed in patients infected with Middle East respiratory syndrome coronavirus (MERS-CoV) (Lau et al., 2013). Further, in the early phase of the cytokine storm, immune cells and cytokines may be clustered in the pulmonary lesions, resulting in a decrease in IL-6 levels in the peripheral blood. Consequently, we observed a mild decline occurred in IL-6 serum levels over the initial 10–20 days of the clinical course (**Figure 4**). In the management of SARS patients, dynamic changes in IL-6 levels have been shown to occur synchronously with the changes in radiographic scores (Chien et al., 2006). Moreover, a similar association between IL-6 levels and CT scores was observed in COVID-19 patients over a period of 20–30 days (**Figure 5**). The decrease in IL-6 levels occurred earlier than the increase in CT scores that reflected the progressive deterioration of lung lesions (**Figure 5**). The disparity in the two aforementioned scores was more obvious in the elevated IL-6 group. Thus, the decreasing trend of IL-6 levels might be predictive of the imminent worsening of lung injuries. In addition, the good predictive capability of IL-6 for disease severity and prognosis in COVID-19 patients, assessed by the ROC curves (AUC > 0.75; **Figure 3**), provided further evidence regarding its predictive function.

Nonetheless, this study has several limitations. First, due to the programmatic and retrospective nature of this study, we could not measure serum IL-6 levels or assess CT images on the same day. Second, the size of the study sample was modest, and there was inadequate statistical power to detect whether there was a differential effect among the mild, severe, and critical COVID-19 cases. Third, as this was a retrospective study, data on all parameters were not available for all patients, and data regarding in-hospital medications may not have been fully recorded. Some patients were transferred from other hospitals and might not have received uniform treatment in the early stages of COVID-19. Fourth, testing for IL-6 levels was performed at different time intervals for each patient. Hence, diagnostic bias may have resulted from the increased number of tests performed in patients with high IL-6 levels. Fifth, we were unable to retrieve pre-hospital self-medication data from the in-hospital electronic records, considering the emergent circumstances of the COVID-19 pandemic. Sixth, median age was different between the elevated IL-6 group and the normal IL-6 group that may have caused bias in the analyses of different laboratory parameters, although we adjusted for age in our multivariate analysis. Finally, the use of some medications, such as immunomodulators used for suppressing an overactive cytokine response may have influenced the experimental results. There may have been unknown interactive effects between medications and IL-6.

## Conclusion

The results from this study need to be interpreted considering other potential and residual confounders. It is likely that a higher IL-6 level is an independent risk factor for in-hospital severity and mortality in Chinese COVID-19 patients, and it might be a potential predictor of lung injury in these patients. The findings of this study provide novel research insights that may guide the early therapeutic intervention for COVID-19 patients, such as dietary interventions (Messina et al., 2020) and medication with IL-6 receptor blockers (Zhang et al., 2020). However, cohort studies, prospective studies, and RCTs are required to further validate the impact of these findings and the early therapeutic strategies.

## Data Availability Statement

The raw data supporting the conclusions of this article will be made available by the authors, without undue reservation.

## Ethics Statement

The studies involving human participants were reviewed and approved by Research Ethics Committee of the Zhongnan Hospital of Wuhan University. Written informed consent for participation was not required for this study in accordance with the national legislation and the institutional requirements.

## Author Contributions

ZL, JL, DC, RG, and WZ designed the study, collected and analyzed the data, and wrote the manuscript. SC, YH, JH, WL, and ML collected and reviewed the clinical, laboratory, and radiological data. ZL, JL, and DC performed statistical analyses. RG and WZ reviewed, interpreted, and checked the clinical data. LG, XWa, and XWu wrote the manuscript and provided valuable suggestions for the study design and data analysis. LG, XWa, and XWu contributed equally, designed the project, edited the manuscript, and supervised the study. All authors contributed to the article and approved the submitted version.

## Conflict of Interest

The authors declare that the research was conducted in the absence of any commercial or financial relationships that could be construed as a potential conflict of interest.

## Supplementary Material

Figure S1 Design schematic of the study.

## Abbreviations

ARDS, acute respiratory distress syndrome; AUC, area under the curve; CI, confidence interval; COVID-19, coronavirus disease 2019; CT, computed tomography; GGO, ground-glass opacity; HR, hazard ratio; Ig, immunoglobulin; IL-6, interleukin-6; INR, international normalized ratio; MERS-CoV, Middle East respiratory syndrome coronavirus; OR, odds ratio; ROC, receiver-operating characteristic; SARS, severe acute respiratory syndrome coronavirus; SARS-CoV-2, severe acute respiratory syndrome coronavirus 2.
