# Supplementary figures and images for: Dynamic Interleukin-6 Level Changes as a Prognostic Indicator in Patients With COVID-19
Source: Front Pharmacol. 2020 Jul 17;11:1093. doi: 10.3389/fphar.2020.01093 (PMC7379481; doi:10.3389/fphar.2020.01093)

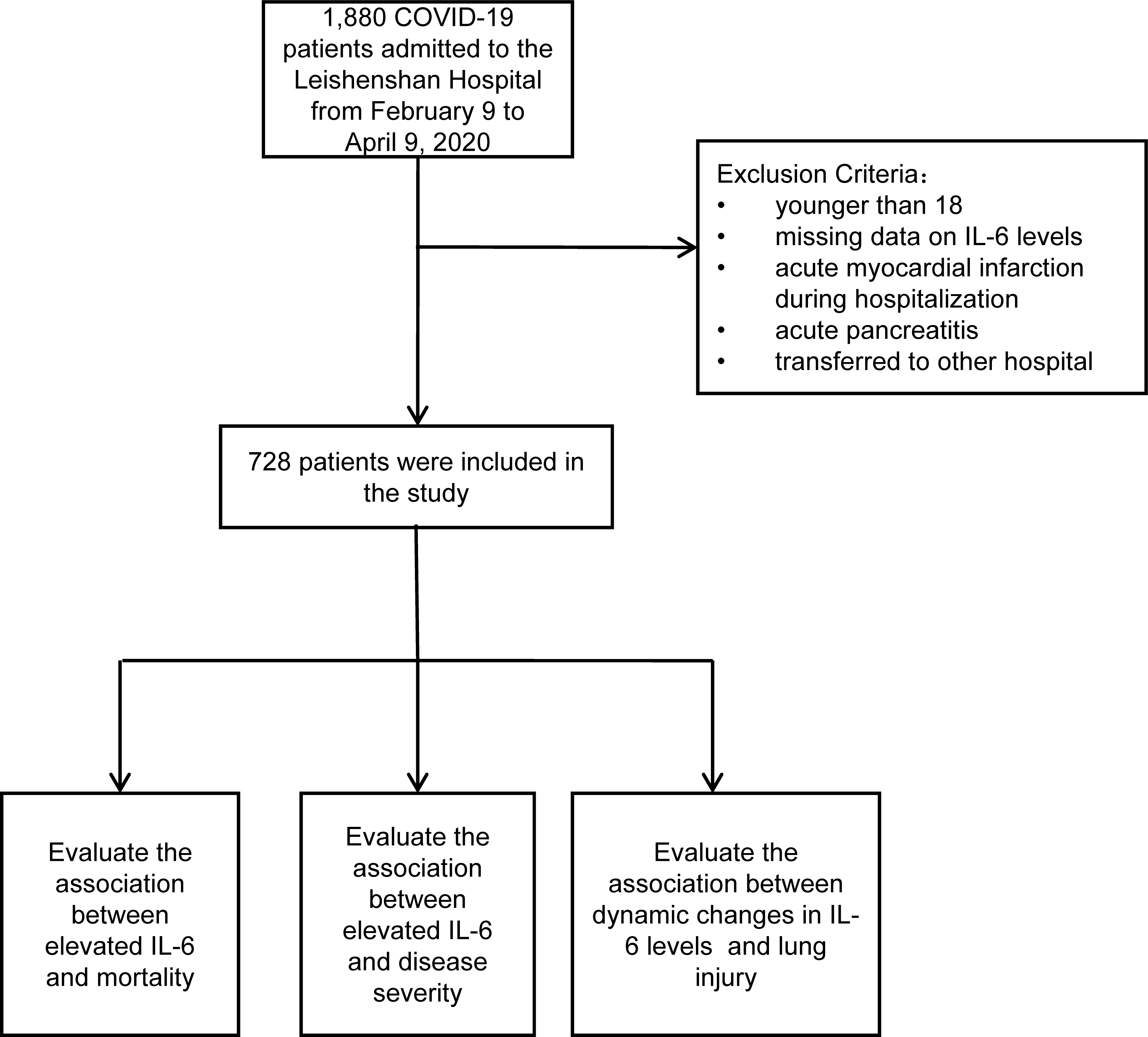

Supplement: Figure S1 — Design schematic of the study. [file Image_1.tif]
